# Scoping Review: Evidence-Based Assessment of Reactive Aggression in Children

**DOI:** 10.1016/j.jaacop.2023.08.005

**Published:** 2023-09-09

**Authors:** Joshua A. Langfus, Eric A. Youngstrom, Chase M. DuBois, Robert L. Findling, Ekaterina Stepanova

**Affiliations:** a University of North Carolina at Chapel Hill, Chapel Hill, North Carolina; b Helping Give Away Psychological Science, Chapel Hill, North Carolina; c Virginia Commonwealth University, Richmond, Virginia

**Keywords:** aggression, rating scales, reliability and validity, children, assessment

## Abstract

**Objective:**

Severe reactive aggression poses a major mental health challenge for many families. A lack of validated instrumentation for assessing young children may present a barrier to more effective clinical assessment and treatment. This scoping review evaluates tools currently used in clinical research to assess aggressive behavior, and identifies gaps in the evidence base for their use in children under the age of 12 years. Measures were evaluated through an evidence-based assessment framework to support clinical decision making.

**Method:**

A comprehensive review of registered clinical trials targeting childhood aggression in the US identified relevant instruments; tools cited in 3 recent reviews of related constructs were also coded. Measures included were available in English, contained at least 3 items measuring aggressive behavior, and had at least 1 validation study in children under 12 years of age. Validation studies were identified through structured queries, and information was extracted from full text review of these studies as well as published manuals.

**Results:**

Of 173 candidate measures, 17 met inclusion criteria: 3 broadband and 14 narrow-band. Compared to commercially distributed measures, free instruments that were more targeted to assess aggression nevertheless had poorer norms and fewer validation studies in children under 12 years of age.

**Conclusion:**

Improving instrumentation for assessing reactive aggression would address an urgent clinical need and a gap in current research. More work is needed to validate measures of reactive aggression in children under 12 years of age, especially studies that include non-clinical comparison samples. Here we recommend broad and narrow measures for providers to use in clinical care, emphasizing tools with good psychometric properties and no cost barrier.

Although occasional tantrums and behavior outbursts are developmentally normative for young children, frequent aggressive outbursts after the third year of life are more rare, highly impairing,^1^^,^^2^ distressing to caregivers,^3^ and costly to society.^4^ These behaviors are among the most common reasons that parents seek mental health services,^5^, ^6^, ^7^ and without effective early intervention, more serious outcomes such as worsening of behavior problems and involvement in the criminal justice system become more likely.^2^^,^^3^

One challenge to effective intervention is that there is significant heterogeneity in the presentation of aggressive behavior in childhood. For example, 2 forms of aggressive behavior—reactive aggression (RA) and proactive aggression (PA)—are associated with different courses and severity of illness^8^, ^9^, ^10^ and have been used to identify clinically distinct subgroups.^11^ PA (also called “instrumental” or “predatory” aggression) typically functions to achieve a tangible reward or interpersonal influence and is often associated with conduct disorder. RA, in contrast, describes behavior occurring in response to some proximal aversive stimulus, such as parental limit setting, and is characterized by high levels of expressed negative affect during the outburst.^12^

Another challenge is that RA is not specific to any single diagnosis. It can occur in the context of mood, disruptive behavior, and other disorders.^13^, ^14^, ^15^, ^16^, ^17^ Our recent work has proposed an empirically defined nosology for RA, showing that aggression with impulsivity/reactivity (AIR) may be distinct from other forms of mood and behavior problems and may represent a separate construct.^13^, ^14^, ^15^ Other researchers have proposed creating new disorder categories^18^ or augmenting existing ones, potentially using aggression as a marker of illness severity.^19^ Overall, despite significant advances in understanding RA, much work remains concerning how to assess, characterize, and treat these impairing behaviors.^20^

### Measuring Reactive Aggression

Although many assessments include some questions about aggressive behaviors, we found that there is a gap in tools that specifically assess RA in children. By “assessment,” we mean any tool that a clinician might use to gain information over the course of treatment, such as a questionnaire, interview, or observation paradigm. Because youth with RA often have complex diagnostic presentations, having valid and reliable assessments of RA symptoms could help clinicians characterize aggressive behaviors, identify therapeutic targets, and monitor treatment progress. For example, the co-occurrence of emotion dysregulation and RA can significantly complicate the diagnostic picture. Children who kick, threaten, throw things, and destroy property can certainly experience intense emotions during these episodes; however, not all children who are emotionally dysregulated exhibit aggressive behavior. Furthermore, some children with frequent, impairing behavioral outbursts do not experience pervasively irritable mood—that is, the aggression is reactive, not a symptom of a mood disorder.^13^^,^^14^^,^^21^^,^^22^ The relationships between irritability, aggressive behavior, and mood symptoms is an active area of current research and debate. Regardless of one’s stance on these issues, evaluating empirical claims related to the prevalence of RA with or without inter-episode irritable mood depends on choosing reliable and valid measures to operationalize the relevant constructs.

The lack of validated RA assessments has implications for both treatment research and clinical practice. First, comparing results across studies requires consistent operationalization of phenomena. Lack of consistency in instrumentation may lead to studies targeting different constructs, in turn leading to incomparable results. Second, the ability to detect a meaningful signal in study data depends on psychometric properties of the measures, such as the reliability. Statistical unreliability in measurement tools will attenuate observed treatment effects, even if the intervention is in fact efficacious. From a practitioner’s perspective, additional concerns include the availability of representative norms and statistical bias across relevant social or demographic factors. Both clinicians and parents might want to know how a child’s behavior compares to typical development. This scoping review can help guide clinicians and researchers in choosing appropriate and available measures for clinical care or outcome research, as well as highlight needs for ongoing measurement development.

### The Evidence-Based Assessment Model

Evaluating the utility of a measure depends not only on considering its psychometric properties, but also the context and goals of the assessment.^23^ The evidence-based assessment (EBA)^24^, ^25^, ^26^ approach highlights 3 key phases of assessment during which different types of assessment may be useful: Prediction, Prescription, and Process. During the Prediction phase, broadband screening measures and semi-structured interviewing can guide initial hypothesizing about a patient’s presenting concerns. Highly sensitive measures can alert the clinician to potential areas of follow-up, but may be less useful for narrowing the differential (eg, ruling in/out a mood disorder). In the Prescription phase, narrower assessments are added to differentiate between competing hypotheses. In addition to targeting a more focused range of symptoms, measures suitable for this phase should have high specificity to the target construct to help reduce the chance of false-positive results. Normative data can be useful here to support diagnosis by showing how assessed symptom severity compares to relevant groups. Finally, once an initial case formulation is finalized and treatment has commenced, Process measures help evaluate treatment effectiveness. Shorter measures that assess specific treatment targets or impairing symptoms can be useful, especially when they demonstrate high reliability and sensitivity. For more information about the EBA model, see Hunsley and Mash^24^ and Youngstrom *et al.*^25^ for reviews.

### The Present Study

To our knowledge, no prior work has attempted to identify what assessments of RA have been used in treatment research, nor has any study evaluated the psychometric properties of these instruments. Several recent reviews have examined assessments for the related domain of emotion dysregulation,^27^^,^^28^ and over a decade ago Hubbard *et al.*^8^ presented some measures of RA and PA. However, no recent studies have collected parent- and clinician-report tools for specifically assessing aggressive behavior in young children. Given the broad heterogeneity in the presentations of aggression, the varying perspectives on how to operationalize these behaviors, and the lack of prior reviews of RA measures, a scoping review such as this is helpful for mapping out the tools most likely to be useful for both clinicians in practice and treatment developers. Rather than cataloging every measure used to assess aggression in children, we focus on those written in English and currently used in clinical trials targeting RA in children under 12 years of age. We supplemented measures found here with others included in recent reviews of similar constructs as well as several notable examples that we found by following leads during the search. By looking first at clinical trials and then broadening the focus, we address the research–practice gap and hope to guide future systematic reviews and meta-analyses focused on these measures. Given the clinical overlap between RA and PA, some of the included measures assess a mix of these symptoms; however, all include RA.

Because no other reviews of aggression assessments in this age range exist and because further work is needed to define the conceptual boundaries of RA vs other related constructs such as emotion dysregulation and conduct problems, a scoping review is an appropriate synthesis at this time. For each identified assessment, we provide a qualitative synthesis of reliability and validity evidence as well as other features to support clinical decision making such as the measure’s length, number of informants, and cost. We also evaluate each tool through the lens of the EBA framework, emphasizing suitability across the 3 facets of treatment, namely, Prediction, Prescription, and Process. Finally, we offer recommendations for broadband and narrow-band measures, highlighting tools with good psychometric properties and no cost barriers to use.

## Method

To gain a representative picture of extant RA assessments and to evaluate their measurement properties, a 2-stage approach was used. Further details, including the Preferred Reporting Items for Systematic Reviews and Meta-Analyses for Scoping Reviews (PRISMA-ScR) checklist, can be found online at https://osf.io/q2zb6.

### Selection of Measures

First, we collected the outcome measures used in every registered clinical trial for childhood aggression listed on Clinicaltrials.gov. Trials were included if they had any of the following terms as keywords: “Youth Aggression,” “Impulsive Aggression,” “Aggression Childhood,” “Aggressive Childhood,” or “Aggressive Outbursts.” Trials in this phase were excluded if they (1) did not include children under 12 years of age (including older children was acceptable if children under 12 were also included); (2) focused primarily on bullying or peer victimization; or (3) measured aggressive behavior secondary to a medical concern (eg, cancer). To gain an even broader list of measures, we then added instruments identified in any of 3 recent reviews (2 narrative, 1 systematic) of assessments for mood and behavioral dysregulation.^8^^,^^27^^,^^28^ Finally, we included 4 additional measures that meet the inclusion criteria outlined below and exemplify potentially useful properties but were not identified in clinical trials or review papers.

Measures were included in the psychometric review if they if they met the following criteria: (1) were available in English; (2) were not exclusively peer- or teacher-report; and (3) had subscales related to aggression or conduct problems with at least 3 items measuring verbal or physical aggression, at least 1 of which had to be physical. Verbal aggression comprised any form of yelling, cursing, or threatening other people; physical aggression included any behavior resulting in physical harm to another person, such as hitting, kicking, biting, or throwing objects.^29^

### Evaluation of Measures

After identifying a list of measures, structured searches on Scopus in April 2023 using a standardized search string identified empirical validation studies reporting the psychometric properties of these tools [eg, for the Parent Daily Report measure, the string was TITLE-ABS(“Parent Daily Report”) AND TITLE-ABS(psychometric∗ OR reliab∗ or valid∗)]. If this initial string yielded no results or a high number of false-positive results, the search was repeated with only the name of the measure or with additional logic (search strings for each measure available in Table S1, available online). Title and abstract review then identified validation studies of the target measure. Although validity information was extracted only from studies that met the inclusion criteria (listed above), the number of citations for anchor publications was also extracted from Scopus (unless otherwise noted) to roughly gauge the overall prevalence of the measure in the literature outside of validation studies. We also recorded populations in which the measure had been used (eg, clinical, non-clinical) and translations reported in titles and abstracts of these studies, regardless of whether the study was included in the psychometric review.

Once validation studies were identified, full-text review of these studies was conducted to extract information about reliability, validity, norms, and sensitivity to treatment. This information was extracted from published manuals,^30^, ^31^, ^32^, ^33^, ^34^ reviews,^8^^,^^27^^,^^28^ or book chapters^35^ when those were available. Ratings were applied based on the EBA framework^24^^,^^25^ (summarized in Table 1^36^^,^^37^). A complete bibliography of studies included in this review is available at https://osf.io/er6az/.

**Table 1:** Crossing Psychometric Parameters With the Three Ps of Evidence-Based Assessment (Prediction, Prescription, and Process)

Criterion	Rubric			
	Adequate	Good	Excellent	Too good /too excellent^a^
Norms	Mean and SD for total score (and subscores if relevant) from a large, relevant clinical sample	Mean and SD for total score (and subscores if relevant) from multiple large, relevant samples, at least 1 clinical and 1 nonclinical	Same as “good,” but must be from representative sample (ie, random sampling, or matching to census data)	Not a problem
Internal consistency (Cronbach alpha, split half, etc)	Most evidence shows alphas of 0.70-0.79	Most reported alphas 0.80-0.89	Most reported alphas ≥0.90	Alpha is also tied to scale length and content coverage; very high alphas may indicate that scale is longer than needed, or that it has a very narrow scope
Inter-rater reliability	Most evidence shows kappas of 0.60-6.74, or intraclass correlations of 0.70-0.79	Most reported kappas of 0.75-0.84, ICCs of 0.80-0.89	Most kappas ≥0.85, or ICCs ≥ 0.90	Very high levels of agreement often achieved by re-rating from audio or transcript
Test–retest reliability (stability)	Most evidence shows test–retest correlations ≥0.70 over period of several days or weeks	Most evidence shows test–retest correlations ≥0.70 over period of several months	Most evidence shows test–retest correlations ≥0.70 over 1 year or longer	Key consideration is appropriate time interval; many constructs would not be stable for years at a time
Repeatability^a^	Bland–Altman^36^ plots show small bias, and/or weak trends; coefficient of repeatability is tolerable compared to clinical benchmarks^37^	Bland–Altman plots and corresponding regressions show no significant bias, and no significant trends; coefficient of repeatability is tolerable	Bland–Altman plots and corresponding regressions show no significant bias, and no significant trends; established for multiple studies; coefficient of repeatability is small enough that it is not clinically concerning	Not a problem
Content validity	Test developers clearly defined domain and ensured representation of entire set of facets	As “adequate,” plus all elements (items, instructions) evaluated by judges (experts or pilot participants)	As “good,” plus multiple groups of judges and quantitative ratings	Not a problem; can point out that many measures do not cover all of the *DSM* criteria now
Construct validity (eg, predictive, concurrent, convergent, and discriminant validity)	Some independently replicated evidence of construct validity	Bulk of independently replicated evidence shows multiple aspects of construct validity	As good, plus evidence of incremental validity with respect to other clinical data	Not a problem
Discriminative validity^a^	Statistically significant discrimination in multiple samples; AUCs <0.6 under clinically realistic conditions (ie, not comparing treatment-seeking and healthy youth)	AUCs of 0.60 to <0.75 under clinically realistic conditions	AUCs of 0.75-0.90 under clinically realistic conditions	AUCs >0.90 should trigger careful evaluation of research design and comparison group. More likely to be biased than accurate estimate of clinical performance
Prescriptive validity^a^	Statistically significant accuracy at identifying a diagnosis with a well-specified matching intervention, or statistically significant moderator of treatment	As “adequate,” with good kappa for diagnosis, or significant treatment moderation in more than 1 sample	As “good,” with good kappa for diagnosis in more than 1 sample, or moderate effect size for treatment moderation	Not a problem with the measure or finding per se; but high predictive validity may obviate need for other assessment components
				Compare on utility
Validity generalization	Some evidence supports use with either more than 1 specific demographic group or in more than 1 setting	Bulk of evidence supports use with either more than 1 specific demographic group or in multiple settings	Bulk of evidence supports use with either more than 1 specific demographic group AND in multiple settings	Not a problem
Treatment sensitivity	Some evidence of sensitivity to change over course of treatment	Independent replications show evidence of sensitivity to change over course of treatment	As “good,” plus sensitive to change across different types of treatments	Not a problem
Clinical utility	After practical considerations (eg, costs, respondent burden, ease of administration and scoring, availability of relevant benchmark scores, patient acceptability), assessment data are likely to be clinically actionable^a^	As “adequate,” plus published evidence that using the assessment data confers clinical benefit (eg, better outcome, lower attrition, greater satisfaction), in areas important to stakeholders^a^	As “good,” plus independent replication	Not a problem

Note: Extending Hunsley and Mash^38^ and Youngstrom *et al.*^25^; adapted from Wikiversity contributors, “Evidence-based assessment/Validity”, Wikiversity, June 4, 2021, 16:35 UTC, <https://en.wikiversity.org/w/index.php?title=Evidence-based_assessment/Validity&oldid=2287877> [accessed June 4, 2021] Published under Creative Commons license CC-BY-SA 4.0: https://creativecommons.org/licenses/by-sa/4.0/. AUC = area under the curve; ICC = intraclass correlation coefficient.

a New construct or category. “Not a problem” indicates that it is unlikely for higher levels of this dimension to be misleading in terms of biased research designs or inherent trade-offs.

#### Content Coding

Content coding for each measure involved reviewing the items and identifying whether they included symptoms across the content areas identified above as relevant to RA: verbal aggression, physical aggression, proactive aggression, and affective reactivity. At least 3 aggression items were required for a measure to be included, and at least 1 of the items needed to cover physical aggression (eg, hitting, fighting, throwing objects). Proactive aggression involved aggression to attain a goal or involved deceit or lying. Affective reactivity included having a quick temper and being easily frustrated. Additional details about content coding are available in Supplement 1, available online.

### Psychometrics Coding

The first 2 authors evaluated the content coverage, psychometric properties, and suitability of the tools for each of the 3-phase EBA framework: Prediction, Prescription, and Process.^24^^,^^25^ The multiple domains of validity and reliability were distributed into 1 rating for each category based on the preponderance of the available evidence. Ratings were aligned with the EBA framework^26^^,^^39^: excellent, good, adequate, less than adequate, and not reported (Table 1). A range was reported when there were substantial differences within a category.

Evidence of reliability and validity were reported in aggregate based on the information available. For example, if a study did not report inter-rater reliability, but did include test-retest reliability and internal consistency, the reliability rating was based on the latter statistic. If evidence related to multiple facets of reliability or validity were reported, a range of ratings was provided. Evidence of validity was inconsistently reported across studies and further complicated by the fact that there is no single diagnostic category that adequately captures RA. Correlations with other validated symptom measures were considered evidence of convergent validity; higher correlations (eg, in the range of 0.5-0.7) were considered excellent, middling correlations (0.3-0.5) were considered good, and smaller but still significant correlations were considered adequate.

Norms were evaluated based on size and representativeness. Large (*N* > 800), census-matched samples that reported mean scores with SDs were considered excellent. Similarly sized convenience samples were considered good, and smaller (*N* > 200) convenience samples were considered adequate. Smaller samples were considered if they included relevant clinical populations.

## Results

### Identification of Measures

**Figure 1:**
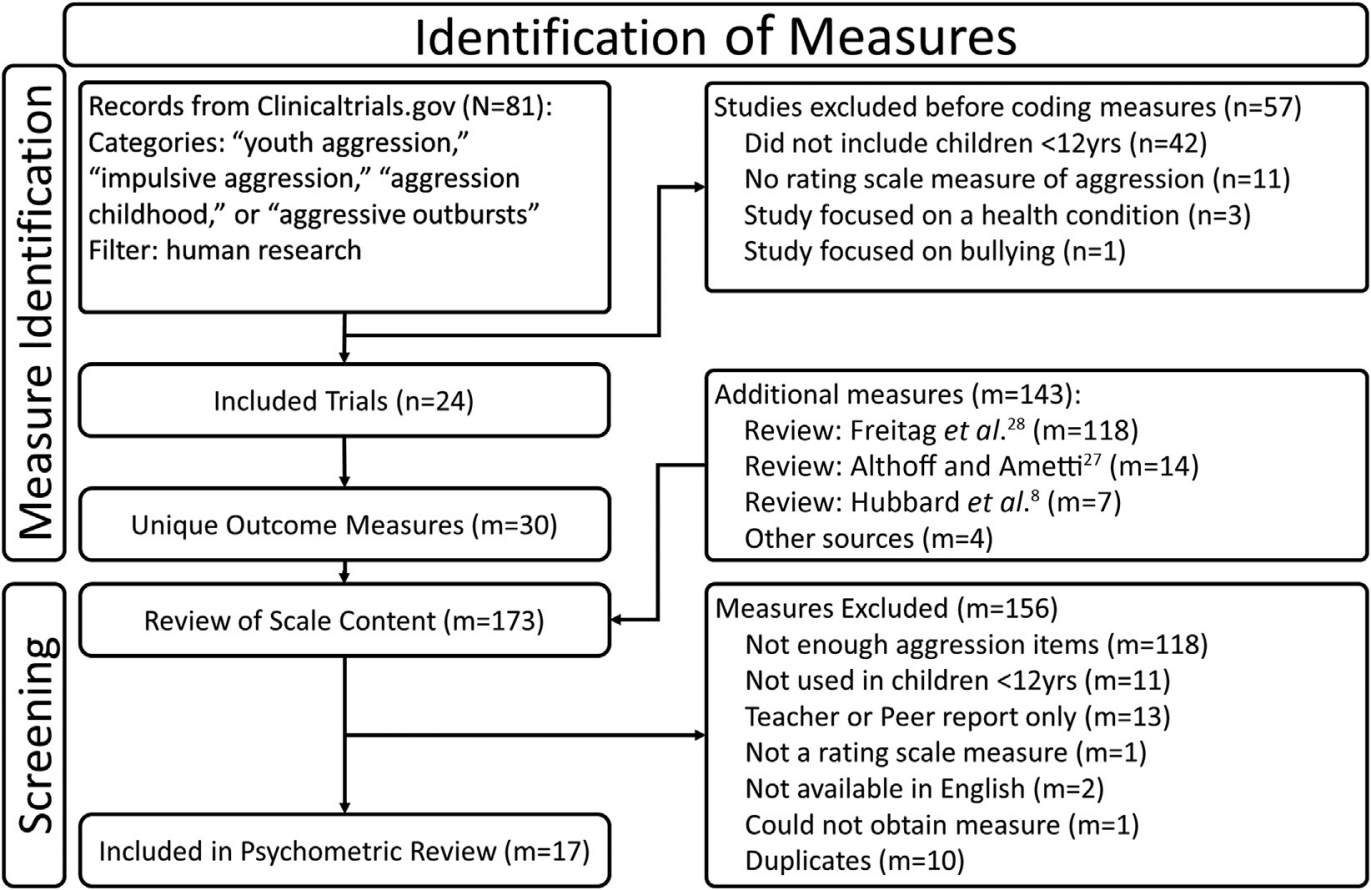
Identification of Measures

We identified 17 measures that met inclusion criteria (Figure 1). First, a comprehensive search found 81 registered clinical trials for aggressive behavior treatments, of which 24 met inclusion criteria. These 24 studies collectively used 30 unique outcome measures. The most commonly used was the Overt Aggression Scale (OAS) or derivatives (ie, Modified OAS [MOAS]; Retrospective-MOAS [R-MOAS]),^40^^,^^41^ appearing in 12 studies, followed by the Child Behavior Checklist, used in 8 studies. Of these 30 measures, 8 met inclusion criteria. To these 8 measures were added an additional 5 that we identified from 3 review papers: a selective review of RA and PA measures^8^ and 2 reviews of emotion dysregulation assessments.^27^^,^^28^ Finally, 4 measures not identified in these searches were also considered. We included the Adjustment Scales for Children and Adolescents (ASCA)^32^ because it takes a uniquely context-sensitive approach to measuring behaviors (discussed below), has excellent norms that define a profile of child with RA, and has been previously used in a large-scale, federally funded mood disorder research study.^42^ We added the Eyberg Child Behavior Inventory (ECBI)^43^ because it is a “highly recommended” measure listed in a recent chapter on conduct problems.^35^ Finally, the Disruptive Behavior Diagnostic Observation Schedule (DB-DOS)^44^ and Parent Daily Report (PDR)^45^ were included because they represent alternative approaches to assessment. The DB-DOS is a diagnostic observation schedule and is conceptually aligned with a questionnaire that we included in the review (see below). The PDR is a clinician-administered phone interview and one of the few with evidence of sensitivity to treatment response.

Overall, we identified 173 unique instruments. Figure 1 illustrates the identification and screening process. Exclusion criteria were evaluated in the order in which they are listed in the figure (top to bottom), and measures were excluded based on the first criterion that they failed. Based on this process, the most common reason for exclusion was lack of verbal or physical aggression items (118 measures), followed by not being appropriate for use in children under 12 years of age (11 measures).

A final set of 17 measures met inclusion criteria for the review of psychometric evidence. These included 3 broadband measures with scales measuring aggressive behavior: the Adjustment Scales for Children and Adolescents (ASCA),^32^ Behavioral Assessment System for Children, 3rd edition (BASC-3),^30^ and the Achenbach System of Empirically Based Assessment (ASEBA)^31^ scales. All 3 of these are commercially distributed. A total of 14 narrow-band measures were identified, including 5 commercial tools and 9 non-commercial measures. Of these, 1 measure explicitly granted permission for use in research: the Buss-Perry Aggression Questionnaire (BPAQ)^46^ and the others have been used in research to varying degrees with unknown copyright status. These narrow-band assessments target 5 related content domains: aggressive behavior, temper outbursts, disruptive behavior, anger expression, and emotion regulation. Most of the measures were questionnaires except for 3: the PDR, a parent-report checklist reported to a clinician over the phone; the Children’s Agitation Inventory (CAI),^47^ a clinician checklist; and the DB-DOS, a clinical diagnostic observation paradigm. The Impulsive Aggression (IA) Diary^48^ has an app-based version in addition to a paper form for parents to fill out daily.

### Evaluation of Identified Measures

**Figure 2:**
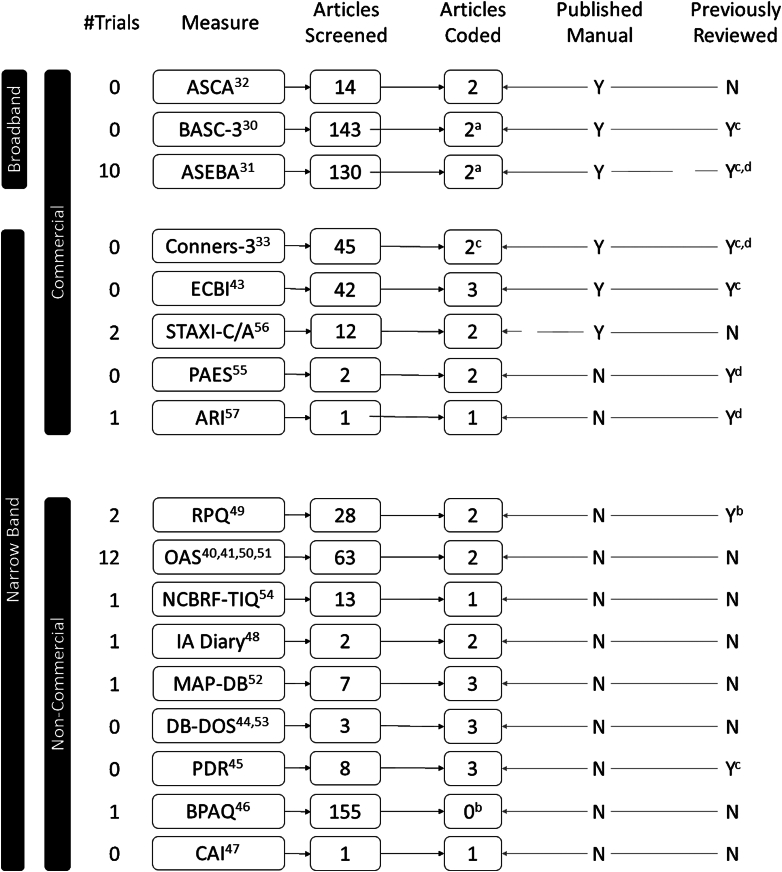
Flow Diagram Depicting Validation Study Search and Data Extraction **Note:** Y = Yes; N = No; 2^a^= Info extracted from manual and previous review only; 0^b^= No studies in children 12 yrs and younger; Y^c^ = rated in Freitag et al.^28^; Y^d^ = rated in Hunsley & Mash Guide to Assessments that Work, 2^nd^ Edition.^35^

Searches for validation studies yielded varying numbers of studies across measures. For research measures, most yielded a small number (no more than 3) of includable validation studies (Figure 2^30^, ^31^, ^32^, ^33^, ^40^, ^41^, ^43^, ^44^, ^45^, ^46^, ^47^, ^48^, ^49^, ^50^, ^51^, ^52^, ^53^, ^54^, ^55^, ^56^, ^57^). Table 2^49^, ^50^, ^51^, ^52^, ^53^, ^54^, ^55^, ^56^, ^57^ summarizes information extracted. The broadband, commercially distributed tools performed the best overall. The majority of instruments (12 of 17) had good to excellent reliability and validity, with 5 exceptions: the CAI, for which no validity or reliability data are available; the Pediatric Anger Expression Scale (PAES-III),^55^ which was rated adequate in these categories; the Anger Response Inventory (ARI),^57^ which had good validity and adequate reliability; the IA Diary, which had adequate reliability; and the BPQA, for which we could find no validation studies in children under 12 years of age. About half of the measures (9 of 17) had any data on sensitivity to treatment in children under 12 years of age, and only 5 rated good or better.

**Table 2:** Measures for Assessing Reactive Aggression in Children Under 12 Years of Age

Measure	Adjustment Scales for Children and Adolescents (ASCA)^32^	Behavior Assessment System for Children, 3rd ed (BASC-3)^30^	Achenbach System of Empirically Based Assessment (ASEBA)^31^	Reactive-Proactive Aggression Questionnaire (RPQ)^49^	Overt Aggression Scale (OAS)^50^ and Derivatives (MOAS^41^, OAS-M^40^, R-MOAS^51^)
Assessment Target	Broadband	Broadband	Broadband	Aggressive Behavior	Aggressive Behavior
Subscale(s)	ADHD Proactive Aggressive (Provocative) Solitary Aggressive (Impulsive) Oppositional Defiant Diffident Avoidant	Hyperactivity Aggression Attention Problems Anxiety Depression Somatization Withdrawal Atypicality	Social Withdrawal Depressed Sleep Problems Somatic Problems Aggressive Destructive	Reactive Proactive	Verbal Aggression Physical Aggression Against Objects Physical Aggression Against Self Physical Aggression Against Others
Licensing	Not free (out of print)	Not free	Not free	Unknown (not marketed)	Unknown (not marketed)
Informants	Parent Teacher	Parent Teacher Self	Parent Teacher Self	Self	Clinician observation (OAS, MOAS) Clinician interview (OAS-M) Parent (R-MOAS)
Target Ages	5-17 y	2 y and up (parent and teacher)	2 y to adult	Children 8 y and up	6-12 y (R-MOAS) Adults (other versions)
Length of measure	156 items	175 items	100 items (Preschool) 113 items (Parent) 113 items (Teacher) 112 items (Self)	23 items	16 items (R-MOAS, MOAS) 20 Items (OAS-M)
Language(s)	English	English, Spanish, French	>110 languages	English, Spanish (Peru), German, Serbian, Dutch, Mandarin, Portuguese (Portugal), Italian, Turkish, Polish, Chinese	English, Italian, Spanish, Mandarin Chinese
Anchor pub cites	90	1952^a^	5641 (Preschool Manual)^a^ 14058 (School-age Manual)^a^	1017	998
Content					
Physical Aggression	Yes	Yes	Yes	Yes	Yes
Verbal Aggression	Yes	Yes	Yes	Yes	Yes
Affective Reactivity	Yes	Yes	Yes	Yes	Yes
Impulsivity	Yes	Yes	Yes	No	No
Proactive Aggression	Yes	Yes	Yes	Yes	No
Response anchor type	Choose All that Apply	Frequency	Severity	Frequency	Choose All that Apply (OAS), Frequency (MOAS and R-MOAS)
Psychometric properties					
Overall norms quality	Excellent	Excellent^b^	Excellent^b^	Adequate	Less than adequate
Reliability evidence	Excellent	Adequate to excellent^b^	Adequate to excellent^b^	Good to excellent	Good
Validity evidence	Good	Good to excellent^b^	Good to excellent^b^	Good	Excellent
Sensitivity to treatment	Not reported	Good	Adequate	Not reported	Adequate
Populations	Non-clinical Clinical (ADHD) Clinical (learning disorder)	Non-clinical Clinical (autism) Clinical (ADHD) Clinical (neurodevelopmental disorders) Clinical (PTSD)	Non-clinical Clinical (conduct disorder) Clinical (ODD) Clinical (forensic) Clinical (bipolar) Clinical (ADHD) CBCL only: Clinical (eating disorders) Clinical (externalizing disorders) Clinical (neurodevelopmental disorders) NSSI (non-clinical)	Non-clinical Clinical (forensic)	Clinical (inpatient) Clinical (Neurodevelopmental disorders) Clinical (ADHD and impulsive aggression) Clinical trials for aggression treatments
Most appropriate for: Prediction, Prescription, Process	Prediction Prescription	Prediction	Prediction	Prescription Process	Prediction Process

Impulsive Aggression Diary (IA Diary)^48^	Children’s Agitation Inventory (CAI)^47^	Multidimensional Assessment of Preschool Disruptive Behavior (MAP-DB)^52^	Disruptive Behavior Diagnostic Observation Schedule (DB-DOS)^44^^,^^53^	Parent Daily Report (PDR)^45^
Temper Outbursts	Temper Outbursts	Disruptive Behavior	Disruptive Behavior	Disruptive Behavior
Aggressive Behaviors	Low Anger Intermediate Anger High Anger Low Distress High Distress	Temper Loss Noncompliance Aggression Low Concern for Others	Behavior Regulation Anger Modulation	Total Problem Behavior Total Stress
Unknown (not marketed)	Unknown (not marketed)	Unknown (not marketed)	Unknown (not marketed)	Unknown (not marketed)
Parent	Clinician	Parent	Clinician	Clinician Parent
6-16 y	4-12 y	3-5 y	Preschool	1.5-3 y (PDR-T) 4-10 y (PDR)
15 items	31 items	111 items	10 tasks 21 codes ∼50 min	34 items
English	English	English, Spanish	English	English
3	69	128	59	260
Yes	Yes	Yes	Yes	Yes
Yes	Yes	Yes	Yes	Yes
Yes	No	Yes	Yes	Yes
No	No	No	Yes	No
No	No	Yes	No	No
Behavior present/absent	Behavior present/absent	Frequency	Severity	Present/absent, last 24 h
Less than adequate	Less than adequate	Good	Adequate	Less than adequate^b^
Adequate	Not reported	Excellent	Good	Adequate to excellent^b^
Good	Not reported	Excellent	Excellent	Adequate to excellent^b^
Not reported	Not reported	Good	Adequate	Not reported
Clinical (disruptive behavior)	Clinical (inpatient)	Non-clinical (epidemiological)	Non-clinical Clinical (high behavior problems) Clinical (low behavior problems) Clinical (ADHD) Clinical (autism)	Non-clinical Children adopted from foster care Children adopted internationally
Prediction Progress	Process	Prescription	Prescription	Process

Eyberg Child Behavior Inventory (ECBI)^43^	Conners, Third Edition (Conners 3)^33^	Nisonger Child Behavior Rating Form - Typical IQ Version (NCBRF-TIQ)^54^	Pediatric Anger Expression Scale, 3rd Edition (PAES-III)^55^	State Trait Anger Expression Inventory– Child and Adolescent (STAXI-C/A)^56^	Anger Response Inventory (ARI)^57^
Disruptive Behavior	Disruptive Behavior	Disruptive Behavior	Anger	Anger	Emotion Dysregulation
Intensity Problem	Inattention Hyperactivity/Impulsivity Learning Problems Executive Functioning Defiance/Aggression Peer/Family Relations	Positive Social Conduct Problems Oppositional Behavior Hyperactive Withdrawn/Dysphoric Overly Sensitive D-Total ADHD-Total	Anger Out Anger Suppression Anger Reflection Anger Control	State Anger State Anger–Feelings State Anger–Expression Trait Anger Trait Anger–Temperament Trait Anger–Reaction Anger Expression–Out Anger Expression–In Anger Control	Anger Arousal Intentions Maladaptive Responses Adaptive Behaviors Escapist-Diffusing Responses Cognitive Reappraisals Long-Term Consequences
Not free	Not Free	Unknown (not marketed)	Not free	Not free	Not free
Parent Teacher	Parent Teacher Self	Parent	Self	Self	Self
2-16 y	6-18 (parent and teacher report) 8-18 (self report)	5-15 y	9-17 y	9-18 y	9-13 y
36 items	Full length: 99 Short forms: 41 (self and teacher) - 45 (parent)	66 items	15	35	20
>30	English, Spanish, French	English	English	English, Spanish	English
448	171 [a]	30	48	48^a^	119
Yes	Yes	Yes	Yes	Yes	Yes
Yes	Yes	Yes	Yes	Yes	Yes
Yes	Yes	Yes	Yes	Yes	No
Yes	Yes	Yes	No	No	No
No	Yes	Yes	No	No	Yes
Frequency Impairment	Frequency/severity	Behavior frequency Problem severity	Frequency	Frequency Severity	Severity
Good^b^	Excellent^b^	Adequate	Less than adequate	Excellent	Not reported
Adequate to excellent^b^	Adequate to excellent^b^	Excellent	Adequate	Adequate to good	Adequate
Good to excellent^b^	Good to excellent^b^	Excellent	Adequate	Good	Good
Good	Good	Good	Not reported	Not reported	Not reported
Chronic illness Developmental disability	Non-clinical Clinical (ADHD) Clinical (conduct problems) Clinical (neurodevelopmental disorders)	TIQ version: Clinical (disruptive behavior) Non-clinical Original version: Clinical (autism) Clinical (intellectual disability)	Non-clinical Chronic illness Clinical (NSSI) Clinical (internalizing disorders)	Non-clinical Medical conditions Clinical, inpatient Clinical, outpatient Clinical (NSSI) Clinical (internalizing disorders)	Non-clinical
Prescription Process	Prediction Prescription	Prescription	Prescription	Prescription	Prescription

Note: ADHD = attention-deficit/hyperactivity disorder; ODD = oppositional defiant disorder; NSSI = non-suicidal self-injury.

a Citation count from Google Scholar rather than Scopus because of lack of citations indexed on Scopus.

b Ratings from Hunsley and Mash.^35^

#### Norms, Reliability, and Validity

Evidence of validity, reliability, and sensitivity to treatment varied across measures. As a rule, only the commercially distributed tools had large, census-matches samples that included non-clinical comparison groups. Psychometric evidence was good to excellent for the broadband tools (BASC-3, ASEBA, and ASCA), as well as for the ECBI and Conners 3.^33^ As may be expected for broadband measures, these tools did not focus on the nuances of aggressive behavior as specifically as aggression-focused instruments (discussed below); however, they have the advantage of broad content coverage of other relevant symptom domains.

In contrast, the non-commercial measures often relied on smaller, convenience samples for validation. Some free measures (eg, the RPQ) had validation studies in non–English-language versions of the measure, which were excluded. Among the non-commercial measures, only 4 tools had adequate or better norms: the RPQ,^49^ Nisonger Child Behavior Rating Form–Typical IQ version (NCBRF-TIQ),^54^ the Multidimensional Assessment of Preschool Disruptive Behavior (MAP-DB),^44^ and the DB-DOS.^44^^,^^53^

#### Content Coverage

Despite the relative paucity of large-scale validation studies for research-derived measures, these tools were generally more targeted at differentiating RA and PA compared to broadband tools or commercial narrow-band measures. Both the ASEBA and BASC-3 forms include an “Aggression” scale; however, the items mix presentations of aggression. For example, the ASEBA Aggression scale includes symptoms of stubbornness/sullenness, disobedience in school, and mood changes, along with attacking people and frequent screaming. Although potentially useful as a broad screener, grouping these items may mask distinctions important for differentiating RA from PA. The ASCA, which reports symptom profiles rather than scale scores, may be an exception; however, the ASCA is no longer generally available. The anger expression scales (PAES-III and the State-Trait Anger Expression Inventory–Child and Adolescent [STAXI-C/A]^56^) also offered some finer-grained distinctions; however, these did not assess more severe forms of aggression (eg, causing serious injuries to others or threatening violence to adults).

The 3 narrow-band measures most focused on aggressive behavior were the OAS, BPAQ, and RPQ. The OAS is well suited for measuring severe aggressive outbursts, having been developed in inpatient settings and extended to include a parent-report version (the R-MOAS).^58^, ^59^ It has four 4-item scales measuring verbal aggression and physical aggression toward self, others, and objects. Uniquely, it also contains items assessing the frequency of longer (>5 minutes) and shorter (<5 minutes) outbursts. A major limitation, however, is that there is no known psychometric evidence supporting this scoring framework, thus limiting its utility as an outcome measure. The BPAQ is a longer measure (29 items) that also assesses verbal and physical aggression, adding content coverage for PA. Although adult and adolescent studies have shown good overall reliability and validity evidence, there are no validation studies in children under 12 years of age. The RPQ is a 23-item self-report scale distinguishing reactive vs proactive aggression in children 8 years and older, with good to excellent evidence of reliability and validity. Unlike the OAS, the RPQ has adequate norms, but no non-clinical comparison samples. Finally, recent work has identified a “dysregulation profile” for the ASEBA scales^27^^,^^60^; although this is potentially useful, further work is needed to evaluate the clinical utility of these items as a stand-alone scale.

The NCBRF-TIQ is notable for having the broadest content coverage of the non-commercial, narrow-band measures. Like the Conners 3 (and, to a lesser extent, the ECBI), it focuses on disruptive behavior broadly and includes items related to verbal and physical aggression, affective reactivity, and impulsivity. Although the items do not probe as many or as severe RA symptoms as the OAS (eg, causing serious injury, such as loss of consciousness), it includes a composite scale measuring conduct and behavior problems broadly (the D-Total scale). The NCBRF-TIQ has adequate norms and excellent validity and reliability; a very similar version of the scale has also been validated in children with developmental and intellectual disabilities.

Finally, it is worth highlighting the DB-DOS for having good-to-excellent reliability and validity evidence, adequate norms, and good sensitivity to change over time. As a performance-based measure, it may provide greater richness of data and ecological validity than questionnaires. Given its 50-minute format, however, it may be more suited to research than clinical contexts. The MAP-DB may serve as a questionnaire-based substitute, as it was developed by the same research group and targets similar behaviors.

### Measures for Prediction, Prescription, and Process

Broadband measures fit well into the Prediction phase, as the wide content coverage can alert a clinician to potential follow-up areas. The BASC-3 and ASEBA can serve in this role, as they have good psychometric evidence and relevant norms. Unfortunately, there is no non-commercial alternative to these tools. Narrow-band measures suitable in the Prediction phase could include the Conners 3 and NCBRF-TIQ. These scales are more narrowly focused on disruptive behavior than the broadband tools, but are still broad enough to provide evidence about symptoms beyond aggression, and therefore their relative length may be justified.

For the Prescription phase, narrow-band measures that can distinguish between different types of aggression are appropriate. The NCBRF-TIQ and Conners 3 might be helpful for differentiating RA from conduct problems. Of all measures, the RPQ focuses most on distinguishing reactive from proactive aggression. As noted above, the DB-DOS may be helpful in cases in which behavioral specificity is especially important and there are no time constraints. Finally, although it does not include as many specific indicators of severe behavior as the R-MOAS, the norms for the RQP make it a useful option.

In terms of Process, the RPQ is relatively brief (23 items), and the availability of norms allows for use of nomothetic benchmarks for clinically significant change. The Parent Daily Report or DB-DOS may be a good choice for measuring within-person change; however these may not be feasible in large-scale studies. Overall, relatively few studies reported statistics relevant to sensitivity to treatment.

## Discussion

This review highlights gaps in the assessment landscape, and helps bridge research and practice by identifying the tools currently used to evaluate symptoms of RA. We reviewed 173 measures and identified 17 assessments appropriate for use in children under 12 years of age. Focusing on measures for this age range addresses a growing need in both research and practice.^20^ Similar reviews of assessments of emotion dysregulation have helped characterize the broad ways in which constructs are operationalized, guiding theory and treatment research.^27^^,^^28^ Although a systematic review fell outside the current scope, this scoping review takes a valuable step toward identifying promising measures of aggressive behavior, laying the groundwork for future quantitative syntheses.

There are 3 main take-aways from this review. First, well-validated commercial measures include some items broadly related to aggressive behavior and are likely to be effective in the Prediction phase of treatment. Having a free alternative would be ideal; unfortunately, the freely distributed Strengths and Difficulties Questionnaire (SDQ)^61^ did not contain sufficient verbal or physical aggression items to be included. For now, the BASC-3 and ASEBA are likely to see continued widespread use in clinical settings.

Second, few narrow-band measures targeting aggressive behavior exist, and those that do lack high-quality norms that include non-clinical samples. During our review, we found that some measures with more extensive validation data in adolescents did not have the same degree of evidence for use in children under 12 years of age. For instance, the Peer Conflict Scale^62^ was excluded for this reason, despite having content relevant to reactive and proactive aggression. Furthermore, although variations of the OAS have been used in many clinical trials, there has yet to be published a large-scale validation study of this instrument. Nevertheless, tools that are more specific in terms of content coverage (the OAS, RPQ, and BPAQ) are generally free to use.

Third, there is a dearth of measures suitable for progress tracking, and very few validation studies reported treatment sensitivity data. Some report test–retest or inter-rater reliability; however these are complementary constructs: a measure must be reliable to pick up a signal, but it must also be tuned to the right “station” (ie, the symptoms expected to respond to treatment). Some treatment developers make study data available on platforms such as the Yale Open Data Access (YODA) project, meaning that there may be exciting opportunities for measurement development in the form of secondary analyses.

The current results suggest several implications for planning assessment batteries sensitive to symptoms of aggressive behavior in young children. In the Prediction phase, providers already using commercial broadband tools may consider choosing the BASC-3 or ASEBA scales, as these are highly reliable, offer multiple informant scales, and have at least some items measuring both RA and PA. For narrow-band tools in the Prescription phase, clinicians may consider the NCBRF-TIQ if severe aggression is not expected and if a broader picture of impulsivity, conduct problems, and social–emotional functioning is desired. For a briefer tool, the 16-item R-MOAS provides a comprehensive picture of RA toward self and others, although it does not currently have norms. If the referral question involves differentiating between reactive vs proactive behaviors, the RPQ may be a good choice. It is relatively brief, has been used in forensic settings, and may help distinguish between oppositional defiant symptoms and more severe conduct problems. For the Process phase, there currently is no ideal measure for tracking treatment progress. The R-MOAS may be helpful here, as it is behaviorally specific, rates frequency of outbursts, and has good psychometric properties. The RPQ may be similarly helpful, and its norms can be used to compute nomothetic benchmarks for change. Another option might be the PDR; designed as a checklist of behaviors that parents report to the clinician over the phone daily, this tool may be ideal for gathering fine-grained information about changes in symptoms over time or in response to behavioral triggers.

Given that RA and other aggressive behaviors have complex and overlapping etiologies, clinicians may consider building a battery of brief and reliable assessments to provide a more comprehensive picture of behavioral concerns. For instance, brief measures of hypomanic and depression symptoms could aid in assessing for a mood disorder; the Parent General Behavior Inventory 10-Item Mania and 10-Item Depression scales^63^ are free, well-validated options. Furthermore, assessing for attention-deficit/hyperactivity disorder (ADHD) symptoms may help substantiate the reactive nature of aggressive behavior; the SNAP-IV^64^ is a free tool with good psychometric properties. In addition, a measure of disciplinary practices, such as the Alabama Parenting Questionnaire,^65^ may be helpful in identifying parenting dynamics that may be inadvertently reinforcing aggressive behaviors. Finally, it may be helpful to use the clinical interview to explore family history of mood and attention problems as well as any history of trauma or perinatal substance exposure.

Although this review is the first to collect and evaluate measures of RA in children, there are several important limitations to consider. First, we identified measures used in treatment research rather than via a comprehensive literature search. Newer measures or those not used in treatment studies may have been missed. Nevertheless, this review is comprehensive of outcome measures used in registered clinical trials and therefore represents the constructs currently shaping treatment development. We also mitigated the likelihood of omitting a relevant tool by including measures reported in other reviews.

We also limited our search to validation studies using the English-language version of a given tool. Evaluating the validity of cross-cultural and cross-linguistic translations falls outside the scope of the current review, but certainly merits its own investigation on a measure-by-measure basis. At least 1 assessment (the Inventory of Callous-Unemotional traits) cited multi-site, multi-country studies as evidence of validity for the English version. Although this type of evidence may support measure validity, evaluating it exceeds our remit. Furthermore, large-scale non-validation studies were not likely to be discovered by our structures search, and therefore our reported reliability and validity may underestimate the measurement properties of these tools.

Finally, we did not include validity studies focusing on children older than 12 years. Therefore, our findings are informative only in terms of measurement of aggression in children 12 years and younger.

Childhood RA is an important clinical construct that spans multiple diagnostic categories. Identifying reliable and valid assessment tools that capture a range of clinically relevant behaviors helps providers and researchers alike. The current work highlights the need for cost-free, narrow-band assessments validated in children under 12 years of age that can be used to distinguish different types of aggressive behavior and also track treatment progress. Validating existing measures that fit this bill or developing new ones may help match children to more effective treatments, as well as shape clinical research by providing outcome measures sensitive to key treatment outcomes.

Future work should consider the potential unintended impacts of RA measures. Even among assessments for which we found the greatest evidentiary support, few studies reported evidence of validity across demographic variables such as race and sex. Although some measures have been validated in diverse samples, it is rare for formal tests of measurement invariance to be reported. As test developers, it is crucial to examine potential bias, lest these tools end up harming the people whom we aim to serve. This is especially true for the assessment of aggression. For instance, children of color in the US are more likely to be perceived by their teachers as more physically formidable,^66^ and labeling a student as “aggressive” could have an unintended negative impact on students with minoritized identities. By the same token, early identification of children at risk for aggressive behavior may lead to earlier and more appropriate intervention. Useful methods exist to evaluate measurement bias, and these techniques should be used and reported when validating clinical assessments.
