# Supplementary material for: Scoping Review: Evidence-Based Assessment of Reactive Aggression in Children
Source: JAACAP Open. 2023 Sep 9;1(4):246–62. doi: 10.1016/j.jaacop.2023.08.005 (PMC11562532; doi:10.1016/j.jaacop.2023.08.005)
Supplement: Supplement 1 [file mmc1.docx]

To be included in this review, the scale has to have at least three items related to verbal or physical aggression, at least one of which must be a form of physical aggression.

Beyond that, items are coded as follows based on reading the items:

| **Content** | **Description** | **Prototypical Symptoms** |
| --- | --- | --- |
| Physical Aggression | Causing harm to another person or damaging property. | Hitting, kicking, scratching, biting, throwing objects, destroying furniture, fighting |
| Verbal Aggression | Abusive or threatening language directed at another person | Cursing, threatening, shouting, insulting |
| Affective Reactivity | Sudden and intense onset of emotion following an eliciting stimulus (usually negative, but could be positive too) | Short temper, gets angry easily, gets angry when told “no”;(NOT: stays angry for a long time) |
| Proactive Aggression | Aggressive behavior that is premeditated or instrumental (used to attain a goal). | Bullying, fights others for status, vengeful or spiteful |
| Response Anchor Type | How are responses coded? Frequency, Severity, Choose the Best, or something else? If more than one anchor type is used on the scale, list both (e.g., Severity, Frequency) | Frequency: Never, sometimes, often, always  Frequency: 0-1 times, 2-3 times, 4-5 times, 5+ times per week  Severity: not true, sometimes true, often true, always true  Severity: not like me, a bit like me, somewhat like me, a lot like me  Choose the Best: from the options, pick the one that describes you best |

For all the categories except Response Anchor, just indicate “Yes” or “No.” For Response Anchor, indicate the type of anchor.
